# Supplementary material for: Distinctive Expression and Amplification of Genes at 11q13 in Relation to HPV Status with Impact on Survival in Head and Neck Cancer Patients
Source: J Clin Med. 2018 Dec 1;7(12):501. doi: 10.3390/jcm7120501 (PMC6306931; doi:10.3390/jcm7120501)
Supplement: Supplementary file 1 [file jcm-07-00501-s001.pdf]

SUPPLEMENTARY INFORMATION

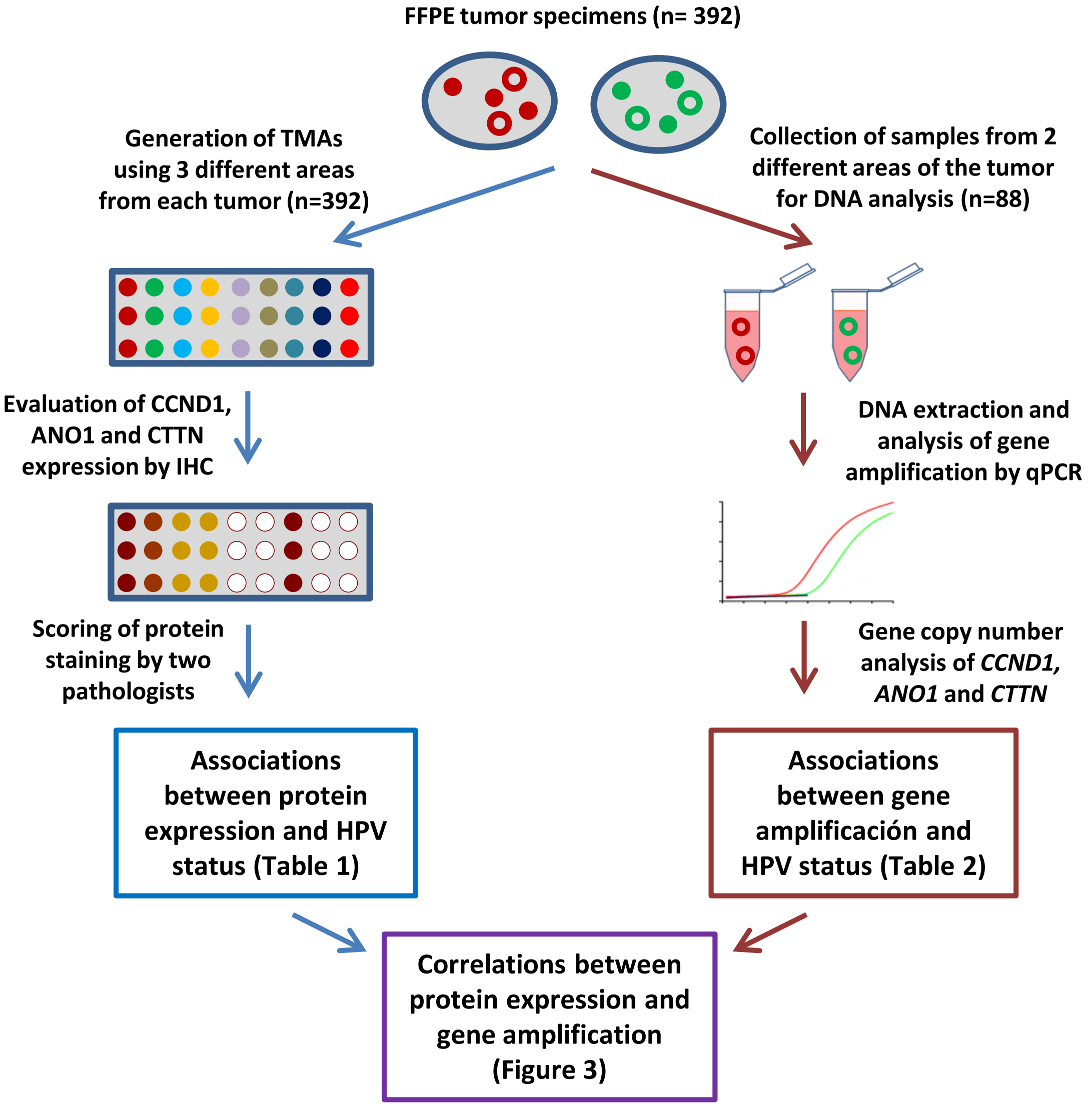

Supplementary Figure S1. Schematic representation of the experimental setup designed to analyze CCND1, ANO1 and CTTN protein expression and gene amplification in relation to HPV status in a cohort of 392 HNSCC patients.

Supplementary Table S1: Clinicopathologic features of the validation cohort of 279 HNSCC patients from the TCGA

	**HPV -**	**HPV +**
**Gender (Age)**		
Male	171 (60.4 +/- 11.8)	32 (58.5 +/- 9.9)
Female	72 (65.2 +/- 13.3)	4 (53 +/- 17.6)
**Tumor site (%)**		
Oral cavity	160 (66)	12 (33)
Oropharynx	11 (4)	22 (61)
Hipopharynx	1 (<1)	1 (3)
Larynx	71 (29)	1 (3)
**Stage (%)**		
I	9 (4)	0 (0)
II	49 (20)	8 (22)
III	64 (27)	3 (8)
IV a	113(47)	23 (64)
IV b	5 (2)	2 (6)
**Tobacco history (%)**		
Life-long non smoker	42 (17)	10 (28)
Current reformed smoker for > 15 yrs	45 (18)	4 (11)
Current reformed smoker for <=15 yrs	69 (28)	12 (33)
Current smoker	80 (33)	10 (28)
**Alcohol history (%)**		
Yes	158 (65)	30 (83)
No	80 (33)	5 (14)
**Total Patients (%)**	243 (87)	36 (13)

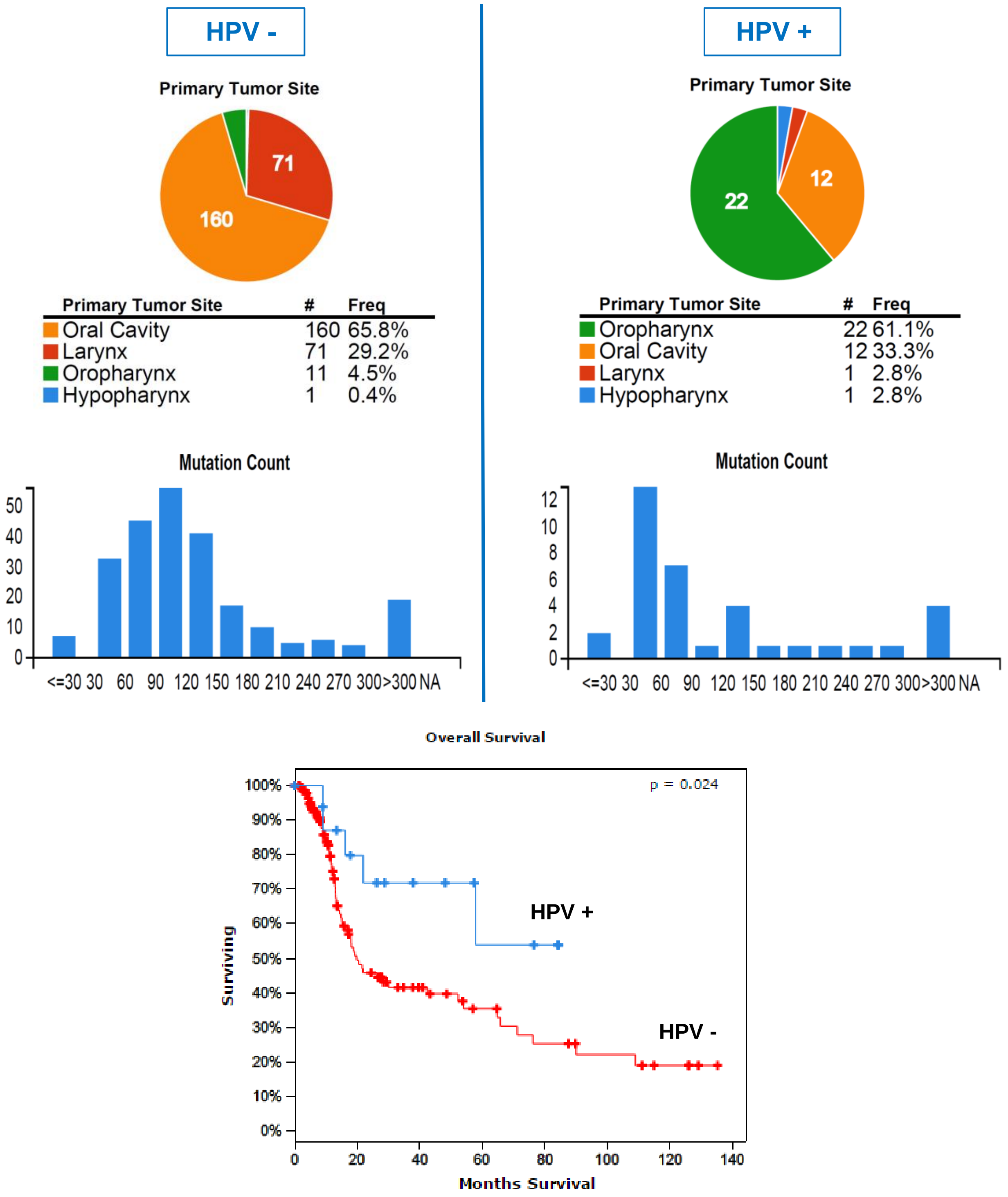

Supplementary Figure S2. Clinical and molecular features of the TGCA cohort of 279 HNSCC patients, according to the HPV status. Schematic representation showing the HPV incidence by HNSCC subsites, mutation frequencies and Kaplan-Meier survival curves (*P* = 0.024, Log-rank test).
